# Determining the Origin of Half-bandgap-voltage Electroluminescence in Bifunctional Rubrene/C60 Devices

**DOI:** 10.1038/srep25331

**Published:** 2016-05-04

**Authors:** Qiusong Chen, Weiyao Jia, Lixiang Chen, De Yuan, Yue Zou, Zuhong Xiong

**Affiliations:** 1 School of Physical Science and Technology, MOE Key Laboratory on Luminescence and Real-Time Analysis, Southwest University, Chongqing 400715, China

## Abstract

Lowering the driving voltage of organic light-emitting diodes (OLEDs) is an important approach to reduce their energy consumption. We have fabricated a series of bifunctional devices (OLEDs and photovoltaics) using rubrene and fullerene (C_60_) as the active layer, in which the electroluminescence threshold voltage(~1.1 V) was half the value of the bandgap of rubrene. Magneto-electroluminescence (MEL) response of planner heterojunction diodes exhibited a small increase in response to a low magnetic field strength (<20 mT); however, a very large decay was observed at a high magnetic field strength (>20 mT). When a hole-transport layer with a low mobility was included in these devices, the MEL response reversed in shape, and simultaneously, the EL threshold voltage became larger than the bandgap voltage. When bulk heterojunction device was examined, the amplitude of MEL curves presented an anomalous voltage-dependence. Following an analysis of the MEL responses of these devices, we proposed that the EL of half-bandgap-voltage device originated from bimolecular triplet-triplet annihilation in the rubrene film, rather than from singlet excitons that formed via an interface auger recombination. This work provides critical insight into the mechanisms of OLED emission and will help advance the applications of bifunctional devices.

Recently, low-cost and flexible organic light-emitting diodes (OLEDs) have made a significant impact on the display and lighting market. However, enhancing their power efficiency remains a major focus within OLED research. Increasing quantum efficiency and reducing the driving voltage are two effective approaches to enhance an OLED’s power efficiency. Ajay K *et al.* have reported an OLED containing rubrene and C_60_, in which the electroluminescence (EL) turn-on voltage was approximately half that of the rubrene bandgap voltage (bandgap energy divided by the electron charge). This emission will be referred to as half-bandgap-voltage EL^1^. This work encouraged significant research efforts into OLED physics^2^,^3^,^4^,^5^. Auger recombination (AR)^1^ and triplet-triplet annihilation (TTA)^2^,^5^ have been proposed as the source of half-bandgap-voltage EL emission; however, the mechanism is currently under debate.

AR and TTA are two common energy up-conversion mechanisms that occur in organic electronics^1^,^2^,^5^,^6^,^7^,^8^,^9^,^10^. AR involves the recombination of an electron-hole pair, which transfers its energy to a third carrier (an electron in C_60_ in this work)^1^. The electron then transfers to the lowest unoccupied molecular orbital (LUMO) of rubrene and recombines with a hole to form an exciton with a singlet-to-triplet ratio of 1:3. As this process consumes two electrons to generate an exciton, the theoretical limit for the quantum efficiency of AR is 12.5%. TTA involves a pair of triplet excitons that annihilate each other, which produces a singlet exciton in the process^11^. The probability of forming triplets in this process is 75%, and therefore the theoretical limit for the quantum efficiency of TTA can reach 62.5% (in addition to 25% singlet excitons)^12^,^13^. So it is essential to understand the origins of half-bandgap-voltage EL for such a huge difference. Time-resolved spectroscopy (photoluminescence or EL) is commonly used to investigate the TTA process in organic semiconducting systems^7^,^12^,^14^. However, it is hard to discern between AR and TTA using time-resolved EL spectroscopy because the exciplex state and triplet exciton of rubrene both have long excited state lifetimes^1^. Therefore, alternative methods must be used.

The energy of a singlet exciton in rubrene is approximately twice that of a triplet exciton (*E*_S_  ≈ 2 *E*_T_). Additionally, both singlet fission (SF)^15^,^16^ and TTA can coexist in OLEDs that contain rubrene^14^. If the half-bandgap-voltage EL originates from AR^1^, the excitons in rubrene must be generated from the recombination of electrons and holes within C_60_ and rubrene, respectively. Thus, if AR is the source of the EL, SF will be dominant over TTA in the device as we have reported previously^13^. However, if the excitons are produced from the transfer of energy from exciplex states^2^, then TTA will be dominant over SF in the device. It has been demonstrated experimentally that both SF and TTA are sensitive to an external magnetic field (*B*), and that their organic magneto-electroluminescence (MEL) responses exhibit diagnostic “fingerprints”^13^,^15^,^16^,^17^,^18^,^19^. To date, MEL has proven to be an effective means to explore the underlying dynamics in organic semiconductors^16^,^19^,^20^,^21^,^22^,^23^,^24^,^25^,^26^. Several models describing MEL have been proposed based on both experimental and theoretical analyses, including intersystem crossing^18^,^22^,^24^,^25^, reverse intersystem crossing^21^, SF^15^,^16^, TTA^11^,^17^ and triplet-charge annihilation (TCA)^20^,^23^. All of these dynamic processes are related to the interactions between different spin-carrying particles (including polaron pairs, bipolarons, excitons or trions) and adhere to the conservation of spin angular momentum. When an applied external magnetic field strength is comparable to the internal strength of the interactive magnetic moments, the evolution of the equilibrium among these particles varies rapidly and then gradually saturates with further increases in the magnetic field strength. Because the internal strength depends on the species of interacting particles and the spatial distance between them, the shape of the MEL curves can be used as a “fingerprint” to distinguish the underlying microscopic mechanisms governing the EL in OLEDs. In this article, we demonstrate that MEL can be used as a convenient method to elucidate the origin of half-bandgap-voltage EL that does not require pre-processing or decomposition of the device. Using this technique, we found that the half-bandgap-voltage EL was caused by TTA in rubrene films, rather than the AR at the interface between rubrene and C_60_.

## Results and Discussion

### Electroluminescent and photovoltaic properties

**Figure 1:**
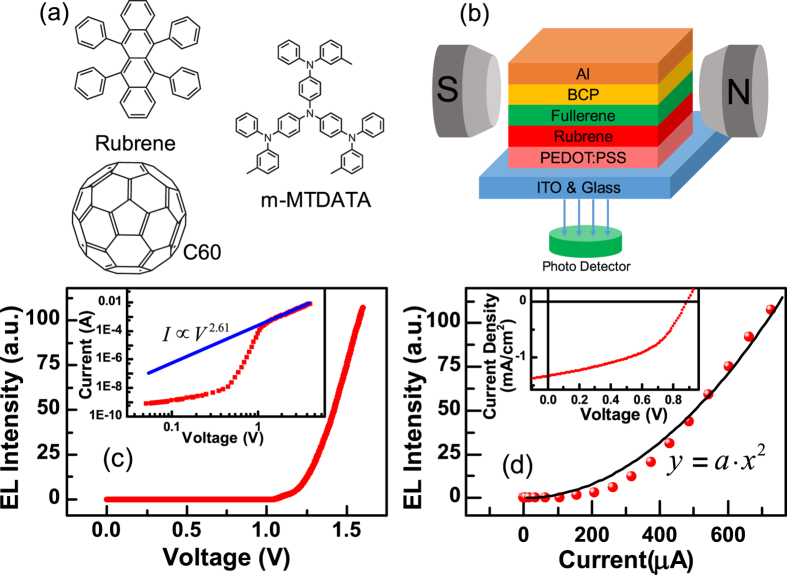
(**a**) Molecular structures of Rubrene, C_60_ and m-MTDATA. (**b**) Schematic of device I under an external magnetic field. (**c**) EL-voltage curve of device I, the inset shows the log-log plot of the current-voltage curve: experimental curve (red) and the fitted line (blue, power function with an exponent of 2.61). (**d**) EL-current curve of device I (red dot) fitted to a quadratic power function (black curve) with a coefficient of a = 1.98 × 10^−4^, the inset shows the current-voltage characteristic under illumination by a laser (405 nm) with an intensity of 20 mW/cm^2^.

The molecular structures of rubrene, C_60_ and m-MTDATA are shown in Fig. 1(a), and a schematic showing a bifunctional half-bandgap-voltage device (device I) being exposed to an external magnetic field is shown in Fig. 1(b). The device structures, fabrication processes and measurements are detailed in the Methods section. The EL-voltage, current-voltage and the photocurrent-voltage characteristics of device I at room temperature (R.T.) are shown in Fig. 1(c,d). The turn-on voltage for the current-voltage and EL-voltage traces were approximately 1.1 V, which is half the value of the bandgap voltage of rubrene (~2.2 V). The current-voltage curve (above the turn-on voltage) was fitted to a power function with an exponent of 2.61, as shown in the inset of Fig. 1(c). This behaviour indicated that space-charge limited current (SCLC)^27^ was dominant in this region. The EL intensity of this device exhibited a quadratic dependence on the current (Fig. 1(d)). The open voltage and short-circuit current of device I were 0.89 V and 1.35 mA/cm^2^, respectively (inset, Fig. 1(d)).

AR that occurs in organic electronics is usually a bimolecular process^8^,^9^. If AR was occurring in device I, this process must require both an exciplex and an electron. As the exciplex state involves two species, this then means that the process of AR actually involves three molecules^1^,^3^,^4^. Therefore the rate of AR should be very small. Klimov *et al.* had reported that the rate for AR in quantum dot system is cubic with respect to the carrier density^10^. However, the experimental curve exhibited a quadratic dependence (Fig. 1(d)). This result was consistent with the emission originating from TTA, because the delayed fluorescence (DF) from TTA is proportional to the square of the triplet density^17^,^19^. Although Pandey has suggested that TTA is responsible for energy up-converted fluorescence^2^, there is still insufficient scientific evidence reported in literatures to demonstrate it.

### MEL response and mechanism of half-bandgap-voltage EL

**Figure 2:**
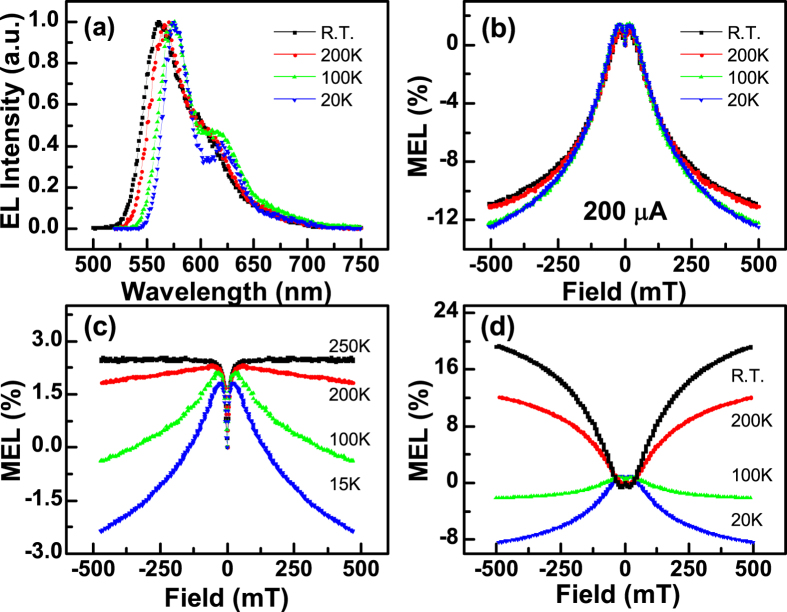
(**a**) EL spectra of device I at R.T., 200 K, 100 K and 20 K. (**b**–**d**) MEL responses at different temperatures for device I, Alq3 based OLED^30^ and conventional rubrene-based OLED^13^, respectively.

The EL spectra of device I at R.T., 200 K, 100 K and 20 K are shown in Fig. 2(a). The EL emission at R.T. exhibited a maximum at 560 nm with a shoulder at 620 nm, which is typical for rubrene emission^13^. MEL is defined as the relative change of EL intensity before and after exposure to an external magnetic field:


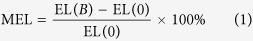


where EL(*B*) and EL(0) are the measured EL intensities with and without an applied magnetic field, respectively. The MEL responses of device I between R.T. and 20 K are shown in Fig. 2(b). A small increase in MEL was observed at low field strength (<20 mT) at each temperature, which was followed by a significant decay at higher field strength (>20 mT). Notably, the amplitude of the MEL response for OLEDs typically varies with temperature and injection current prominently^13^,^25^,^28^,^29^,^30^; however, this behaviour was not observed to any great extent in device I (the MEL curves with different injection currents are shown in Fig. S1), with only a small change (approximately −12%) observed at 500 mT. The typical MEL responses of OLEDs containing Alq_3_ is shown in Fig. 2(c)^30^. The Lorentzian line-shape observed at 250 K resulted from hyperfine fields that were induced by intersystem crossing^18^,^22^. At lower temperatures, the triplet lifetime was increased. As such, DF from TTA made greater contributions to the EL as the temperature was lowered. If TTA was the dominant emission mechanism, the shape of the MEL response should decay at high *B* strength, and the amplitude of this decay should increase as the temperature decreases^17^,^30^,^31^. A comparison of the MEL curve at 15 K shown in Fig. 2(c) with those in Fig. 2(b) indicated that the emission of device I may be derived from TTA; however, further evidence is required to prove this.

The ability of rubrene to undergo SF can be exploited to double the exciton harvesting in photovoltaics^2^,^4^,^16^. A typical MEL response of an OLED that contains rubrene as the active material is shown in Fig. 2(d) 13. The black trace, recorded at R.T., shows a representative SF-dominated MEL response^15^,^16^, that exhibits a small decrease in MEL at low *B* strength (<20 mT) followed by a significant increase in high *B* strength (>20 mT). As the temperature was decreased, the MEL response reversed in shape because the SF within rubrene, which is an endothermic process, was weakened while TTA was enhanced because of the increasing triplet lifetime^16^. A comparison of the curves shown in Fig. 2(b) with other TTA-dominated MEL responses^11^,^17^,^19^,^31^ indicated that this behaviour represented the characteristic response of fluorescence emission derived from TTA. The effects of TTA are just perceptible in the MEL responses at low temperature (Fig. 2(b,c)). However, that fact that evidence of emission from TTA was observed at R.T. in device I, and that the MEL curves did not show any significant change with temperature and injection current needs to be understood. To explain these novel observations, we analysed the excited states and energy-transfer processes within device I.

Rubrene is an extensively studied, hole-transporting organic material with a triplet energy that is approximately half of its singlet energy (E_T_ = 1.14 eV, E_S_ = 2.23 eV, E_S_ ≈ 2 E_T_)^2^. Therefore, one singlet exciton (S_1_) can split into two triplet excitons (T_1_) with the participation of a ground state S_0_ (SF), and two triplet excitons can simultaneously annihilate one another to form a singlet exciton (TTA)^14^. These two processes can be expressed as:


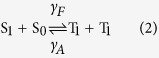


where *γ*_F_ and *γ*_A_ are the rate constants of SF and TTA, respectively. Merrifield’s phenomenological theory^11^,^19^ states that there is an intermediate complex state in equation (2), and it can form 9 collision pair states. The applied external *B* competes with the zero field splitting interactions between two participating excitons. At low *B* strength, the number of pair states ranges from 3 to 6 (these have some singlet character). At high *B* strength, there are only two states with nonzero singlet contribution^19^. Thus, both TTA and SF should show characteristic “fingerprint” shapes in their MEL response because they make either positive or negative contributions to the EL^13^,^16^,^19^.

Under specific exciton generating conditions, the density of singlets and triplets will maintain a dynamic equilibrium by the competition of SF, TTA and other radiative or non-radiative processes. Therefore, the net direction of equation (2) is determined by the following equation:


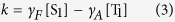


where [S_1_] and [T_1_] are the densities of singlet and triplet excitons in rubrene. When the sign of *k* is positive, the MEL curves will exhibit the characteristic line shape of SF^13^,^15^,^16^, as discussed above (*i.e.* SF is dominant over TTA). However, when the sign of *k* is negative, the MEL curves will exhibit the characteristic line shape of TTA^11^,^17^,^31^, which is opposite to that observed when SF is dominant. The excitons generated in OLEDs that contain rubrene are generally formed from the recombination of free holes and electrons with a singlet-to-triplet ratio of approximately 1:3. These then decay to the ground state through various processes, including photon emission, SF and thermal vibration. These conditions should result in a positive *k* at R.T^13^. However, the MEL curves shown in Fig. 2(b) show the line shape of TTA, which meant that the sign of *k* was always negative for device I. This means that TTA was dominant over SF, even at R.T. This conclusion is contrary to the proposal that electrons can be injected into rubrene via AR at the interface with C_60_^1^. If an electron was activated by AR, it should recombine with a hole in rubrene to form an exciton, which would result in a positive value of *k* and a MEL response with a characteristic SF line-shape at R.T., as shown in Fig. 2(d).

**Figure 3:**
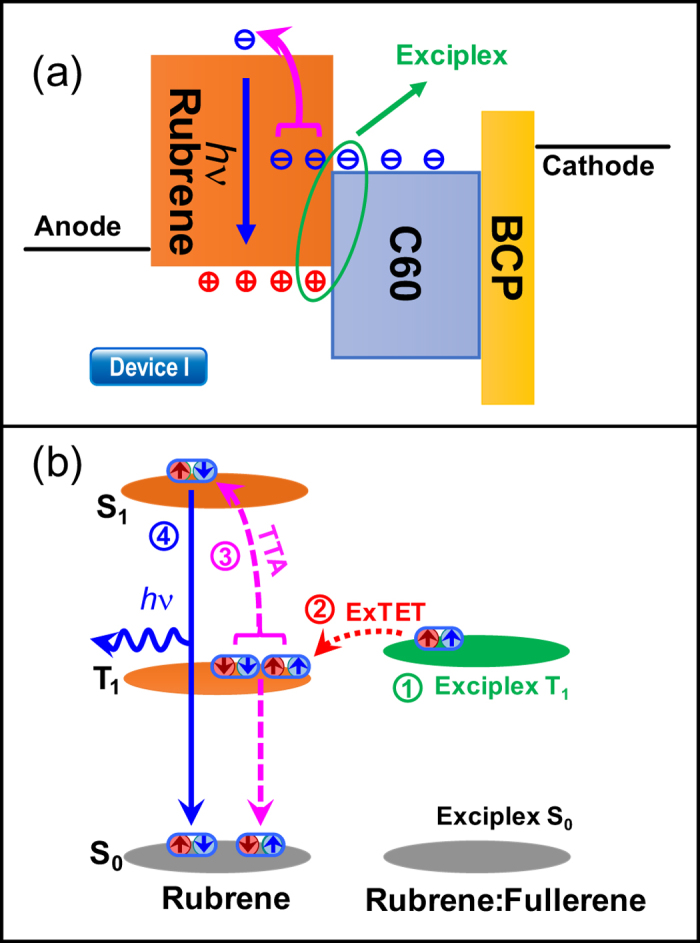
(**a**) Energetics and charge transportation diagram for device I. (**b**) Schematic of energy transfer processes in device I.

These results have led us to propose a four-step mechanism for half-bandgap-voltage EL. 1. Formation of an exciplex at the rubrene: C_60_ interface. The green ellipse in Fig. 3(a) represents the formation of an exciplex between the LUMO of C_60_ and the highest occupied molecular orbital (HOMO) of rubrene. 2. Exciplex-triplet energy transfer (ExTET)^32^,^33^ between two excited states. The energy level alignment of the exciplex and the rubrene exciton is shown in Fig. 3(b). As the energy level of the exciplex is higher than that of the rubrene triplet^1^,^3^,^4^, it can transfer via Dexter energy transfer^34^ (red doted arrow in Fig. 3(b)). 3.The TTA process. The triplets in two adjacent rubrene molecules will generate a singlet exciton (magenta arrow in Fig. 3(b)) via TTA. 4. Photoemission of singlet exciton (blue arrow in Fig. 3(b)). This emission is a form of DF. These four steps elucidate a complete procedure of how half-bandgap-voltage EL is produced.

The large energy barrier that is present between the LUMO of rubrene (3.2 eV) and C_60_ (4.3 eV) should ensure an injection charge limit current (ICLC) regime. However, SCLC was observed (inset, Fig. 1(c)), which closely followed the Mott–Gurney Equation^27^ (blue line in the inset of Fig. 1(c)) and therefore the current showed a quadratic dependence with voltage. This confirmed that the electrons were not driven up into the LUMO of rubrene, but transferred via ExTET to rubrene from an exciplex. This mechanism implies that the EL of device I consisted almost exclusively of DF derived from TTA, which explains why the sign of *k* was negative and why the amplitude of the MEL curves did not change with injection current or temperature.

### Voltage dependence of MEL response in a bulk-heterojunction device

**Figure 4:**
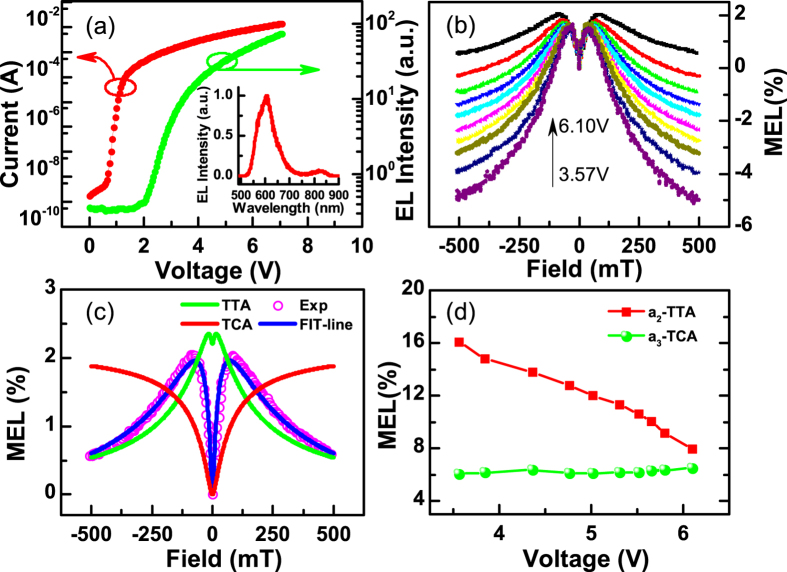
(**a**) Half-log plot of current-EL-voltage curves for device II at R.T., the inset shows the EL spectrum of device II. (**b**) MEL(*B*) responses of device II under different bias voltages at R.T. (**c**) Fitted result for the black curve in (**b**): experiment data (violet circles), TTA fraction (green curve), TCA fraction (red curve) and fitted curve (blue curve). These curves were separately shown in Fig. S2. of supplementary information. (**d**) The value of a_2_ and a_3_ as functions of driving voltage.

If half-bandgap-voltage EL is caused by AR at the interface between rubrene and C_60_, blending these materials should enhance the EL intensity as the interface contact is increased. A bulk heterojunction structure (device II) was fabricated to investigate this hypothesis. The current and EL intensity as functions of voltage, and the MEL response with different voltage are shown in Fig. 4(a,b), respectively. The turn-on voltage for the current-voltage and EL-voltage are 1.2 V and 2.2 V, respectively (Fig. 4(a)). When rubrene and C_60_ were blended (device II), the EL intensity was approximately one order of magnitude smaller than that of device I under the same current density. The EL spectrum of device II exhibited a peak that was consistent with emission from rubrene, with a small shoulder at 850 nm (inset, Fig. 4(a)) which should be resulted from exciplex emission. TTA involves Dexter energy transfer, which is a short range process. Thus, blending the active materials separates the rubrene molecules, which in turn reduces the rate constant *γ*_A_. Therefore, the blending actually reduces the EL intensity rather than enhancing it, as predicted by the AR model.

The MEL curves shown in Fig. 4(b) all contained the “fingerprint” line-shape of TTA, which yielded a negative value of *k* and indicated that DF played a crucial role. However, the increase in the MEL response observed at low *B* strength for device II was more prominent than for device I (Fig. 2(b)). This difference may have been caused by TCA^20^,^23^, in which triplet excitons collide with excessive charge carriers, resulting in non-radiative decay. The applied *B* can perturb the rate of TCA, thereby increasing DF and causing a positive MEL response^20^. The majority of MEL responses can be fitted to either Lorentzian or non-Lorentzian functions^18^,^20^,^21^,^22^,^23^,^35^. Therefore, we fitted our experimental curves to an equation that contained one Lorentzian function and two non-Lorentzian functions with different saturation fields. The equation is as follows:


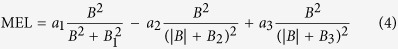


where the first two terms describe the low *B* increase and the high *B* decay in the MEL response to TTA, and the third function describes the MEL response to TCA. We first fitted the curves shown in Fig. 2(b) to the first two terms to determine the pre-factors (a_1_, a_2_) and the saturation fields (*B*_1_, *B*_2_) of TTA. Then, we kept the ratio of a_2_ to a_1_ constant and used the same values of *B*_1_ and *B*_2_, obtained from the TTA analysis, to fit the curves shown in Fig. 4(b). The fits for the MEL curve obtained under a bias of 6.10 V (black line in Fig. 4(b)) are shown in Fig. 4(c) (fitting details are separately shown in Fig. S2. of supplementary information). The saturation fields *B*_1_, *B*_2_ and *B*_3_, determined from the fits, were 9.40 mT, 74.70 mT and 35.00 mT, respectively. The ratio of a_2_ to a_1_ was 8.29. The variation of the pre-factors a_2_ and a_3_ as functions of the driving voltage is shown in Fig. 4(d). The pre-factor a_2_ exhibited a strong negative dependence on the bias voltage, while a_3_ remained almost constant.

When the current density in the TTA-dominated OLEDs was increased by increasing the bias voltage, the decay in the MEL response at high *B* became more pronounced because DF was enhanced over prompt fluorescence (PF) due to the quadratic current dependence of TTA^28^,^31^. However, the negative slope of a_2_ (Fig. 4(d)) indicated that PF increased more than DF as the bias voltage was increased. Plausibly, this anomalous result may have been caused by electrons on C_60_ acquiring energy from the electric field and then recombining directly with holes on rubrene to form a singlet exciton, yielding PF. When the bias voltage was increased, an increasing amount of electrons may have been forced to form singlet excitons directly. Under these conditions, the proportion of PF would increase while the DF would decrease, causing a_2_ to decrease with an increasing bias voltage.

### MEL response of a hole injection-restricted device and the direct charge injection model

**Figure 5:**
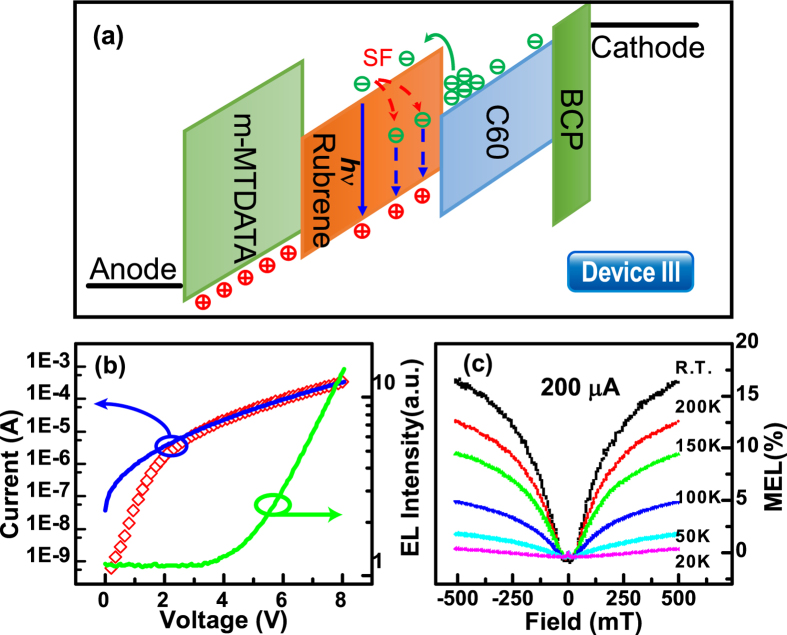
(**a**) Schematics of charge transport within device III. (**b**) Half-log plot of current-EL-voltage curves for device III at R.T., experimental current-voltage (open diamonds), experimental results fitted to the Richardson–Schottky model of ICLC (blue line), experimental EL-voltage (green line). (**c**) MEL (*B*) responses of device III at different temperatures with a current of 200 μA.

We replaced the PEDOT: PSS layer in device I with a hole-transport layer (m-MTDATA) that has a low mobility (device III). An energy-level diagram that shows the charge transport within device III is shown in Fig. 5(a). The hole mobility of m-MTDATA is approximately 1.3 × 10^−5^ cm^2^/Vs^36^,^37^, which is several orders of magnitude lower than that of amorphous rubrene (1 cm^2^/Vs)^38^. The restricted hole injection, caused by the m-MTDATA layer, should reduce the density of exciplex states, which leads ExTET hard to happen and results in the buildup of an excessive amount of electrons. Conversely, the Ohmic contact at the C_60_/cathode interface, combined with the high electron mobility of C_60_ (0.3 cm^2^/Vs^39^), makes that the injection of electrons is more facile than that of holes. The large difference in the LUMO energy levels of rubrene and C_60_ (1.3 eV)^1^ means a high bias voltage is required to drive electrons into the LUMO of rubrene (Fig. 5(a)). Therefore, the turn-on voltage must exceed the bandgap voltage of rubrene, and the current must be ICLC^40^. If the previous conditions are met, then a proportion of electrons will be injected directly into the LUMO of rubrene to form singlet excitons, which results in PF. The decay rate of a singlet exciton is larger than that of an exciplex^41^. Thus, the formation of excitons lowers the proportion of holes available for the formation of exciplex states. A singlet exciton can emit a photon (blue arrow, Fig. 5(a)) or be split into two triplet excitons (red dotted arrows, Fig. 5(a)). Thus, the sign of *k* for device III should be positive and the MEL response should show the “fingerprint” shape of SF.

This analysis was consistent with our experimental results. The turn-on voltages for the current-voltage and EL-voltage traces were approximately 2.2 V and 4.0 V, respectively (In Fig. 5(a)). These values meet the requirement of a driving voltage that is higher than the bandgap voltage of rubrene. The Richardson–Schottky model of ICLC^42^ shows that the current *I* is proportional to a function, given by the following relationship:


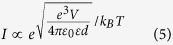


where *e* is the electronic charge, *V* is bias voltage, *ε*_0_ is vacuum permittivity, *ε* is relative permittivity (2.1 for most organic materials), *d* is the thickness of device III (135 nm), *k*_*B*_ is Boltzmann constant and *T* is the temperature of device. At R.T.(300 K), this function can be simplified to a form as follow:


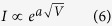


The theoretical value of *a* is 2.76 V^−0.5^, while the experimentally fitted value was 3.21 V^−0.5^ (blue curve, Fig. 5(b)). Thus, the theoretical analysis agrees with our experimental results and confirmed that device III exhibited ICLC. The MEL analysis, shown in Fig. 5(c), presents the “fingerprint” shape of SF as expected. The positive value of *k* indicated that PF dominated the EL; therefore, it was difficult to observe TCA in these MEL responses. The amplitude of the MEL curves decreased with decreasing temperatures. This may have occurred because TTA is enhanced by increased triplet lifetimes and because SF is an endothermic process that weakens^16^ at lower temperatures.

Additionally, we fabricated several other devices with a similar configuration to device I; however, with BCP layers of varying thicknesses. The BCP layer restricted the injection of electrons, which meant a sufficient amount of holes were supplied to form exciplex states with electrons, and subsequently transfer energy via ExTET. The EL of these devices originated from TTA; therefore, their MEL responses exhibited the same TTA ‘fingerprint’ , as shown in Fig. 2(b).

## Conclusion

We have fabricated planar and bulk heterojunction devices using rubrene and C_60_ active layers. The MEL response of the planar heterojunction device (device I) with half-bandgap-voltage threshold EL exhibited the “fingerprint” line shape of TTA, while that of the device that contained a low mobility hole-transporting layer exhibited the reverse shape, consistent with SF. An analysis of the mechanism of MEL allowed us to deduce that half-bandgap-voltage EL originates from the annihilation of two triplet excitons to give a emissive singlet. The triplet excitons formed in these devices were generated via ExTET from the exciplex that was formed between the HOMO of rubrene and the LUMO of C_60_. This proposal is consistent with the work published by Xiang *et al.*^5^. The variation in the amplitude of the MEL curves as a function of applied voltage for the bulk heterojunction device (device II) and the SF ‘fingerprint’ shape in MEL response for the hole-transport restricted device (device III) confirmed that singlet excitons, and thus fluorescence, can be generated either directly or indirectly, *i.e.* through ExTET. Additionally, the generation of singlet excitons via these approaches can be modified by changing the charge injection conditions. These experiment results have expanded the scope of MEL in organic electronics, and importantly have provided a simple method to determine the origin of half-bandgap-voltage EL. This will contribute significantly to the development and advancement of energy-saving bifunctional organic devices.

## Methods

PEDOT:PSS was spin coated onto ITO patterned glass substrates and then annealed for 10 min at 120 °C in 10^−4^ Pa after the ultrasonic baths of deionized water, alcohol and acetone in sequence. The molecular layers used in the devices were grown using an organic molecular beam deposition method under a pressure of 10^−6^ Pa. The planar heterojunction device (device I) had the following the structure: ITO (120 nm)/PEDOT: PSS (40 nm)/rubrene (35 nm)/C_60_ (50 nm)/Bathocuproine (BCP 10 nm)/lithium fluoride (LiF) (1 nm)/Al (120 nm). The bulk heterojunction device (device II) had the following structure: ITO (120 nm)/PEDOT: PSS (40 nm)/rubrene (30 nm)/rubrene: C_60_ (3:1 wt%, 30 nm)/C_60_ (40 nm)/BCP (10 nm)/LiF (1 nm)/Al (120 nm). Device III had the same structure as device I; however, the PEDOT: PSS layer was replaced with an m-MTDATA (40 nm) layer. All devices had an active layer with an area of 2 × 2 mm^2^.

MEL was measured with the samples mounted on the cold finger of a closed-cycle cryostat (Janis CCS-350S) that was located between the poles of an electromagnet (Lakeshore EM647), as depicted in Fig. 1(b). The magnetic field had a maximum strength of 500 mT and was applied parallel to the device surface and measured using a Hall probe GaussMeter (Lakeshore 421) that was placed close to the sample. A Keithley 2400 SourceMeter was used to provide a constant voltage and measure the current. The brightness was determined using a magnetic insensitive silicon photodetector and recorded using a Keithley 2000 apparatus. Further details of the measurements of the MEL response is given in previous publications^13^,^31^. The EL spectra were measured using a SpectraPro-2300i spectrum unit. The photo-current was recorded using a Keithley 2400 while the devices were illuminated with a laser beam (405 nm) with an intensity of approximately 20 mW/cm^2^.

## Additional Information

**How to cite this article**: Chen, Q. *et al.* Determining the Origin of Half-bandgap-voltage Electroluminescence in Bifunctional Rubrene/C60 Devices. *Sci. Rep.* **6**, 25331; doi: 10.1038/srep25331 (2016).

## Supplementary Material

Supplementary Information
