# Supplementary material for: Determining the Origin of Half-bandgap-voltage Electroluminescence in Bifunctional Rubrene/C60 Devices
Source: Sci Rep. 2016 May 4;6:25331. doi: 10.1038/srep25331 (PMC4855215; doi:10.1038/srep25331)
Supplement: Supplementary Information [file srep25331-s1.pdf]

Supplementary Information:

Determining the Origin of Half-bandgap-voltage Electroluminescence in Bifunctional Rubrene/C60 Devices

Qiusong Chen, Weiyao Jia, Lixiang Chen, De Yuan, Yue Zou, Zuhong Xiong*

School of Physical Science and Technology, MOE Key Laboratory on Luminescence and Real-Time Analysis, Southwest University, Chongqing 400715, China
*zhxiong@swu.edu.cn

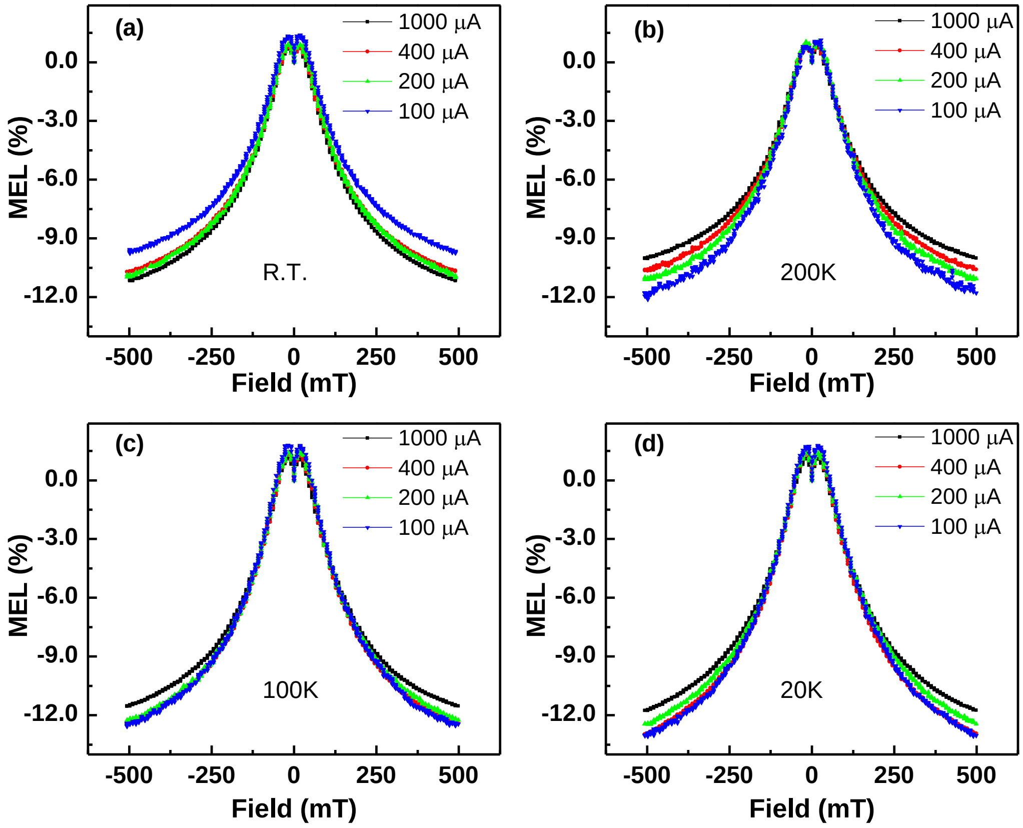

Figure. S1. MEL responses with different injection currents for device I at various temperatures: (a) for R.T., (b) for 200K, (c) for 100K, (d) for 20K. All of these MEL curves showed that the "fingerprint" line shape of TTA, and the amplitude of these MEL curves did not change significantly with both injection current and test temperature, with only a small change (approximately −12%) observed at 500 mT.

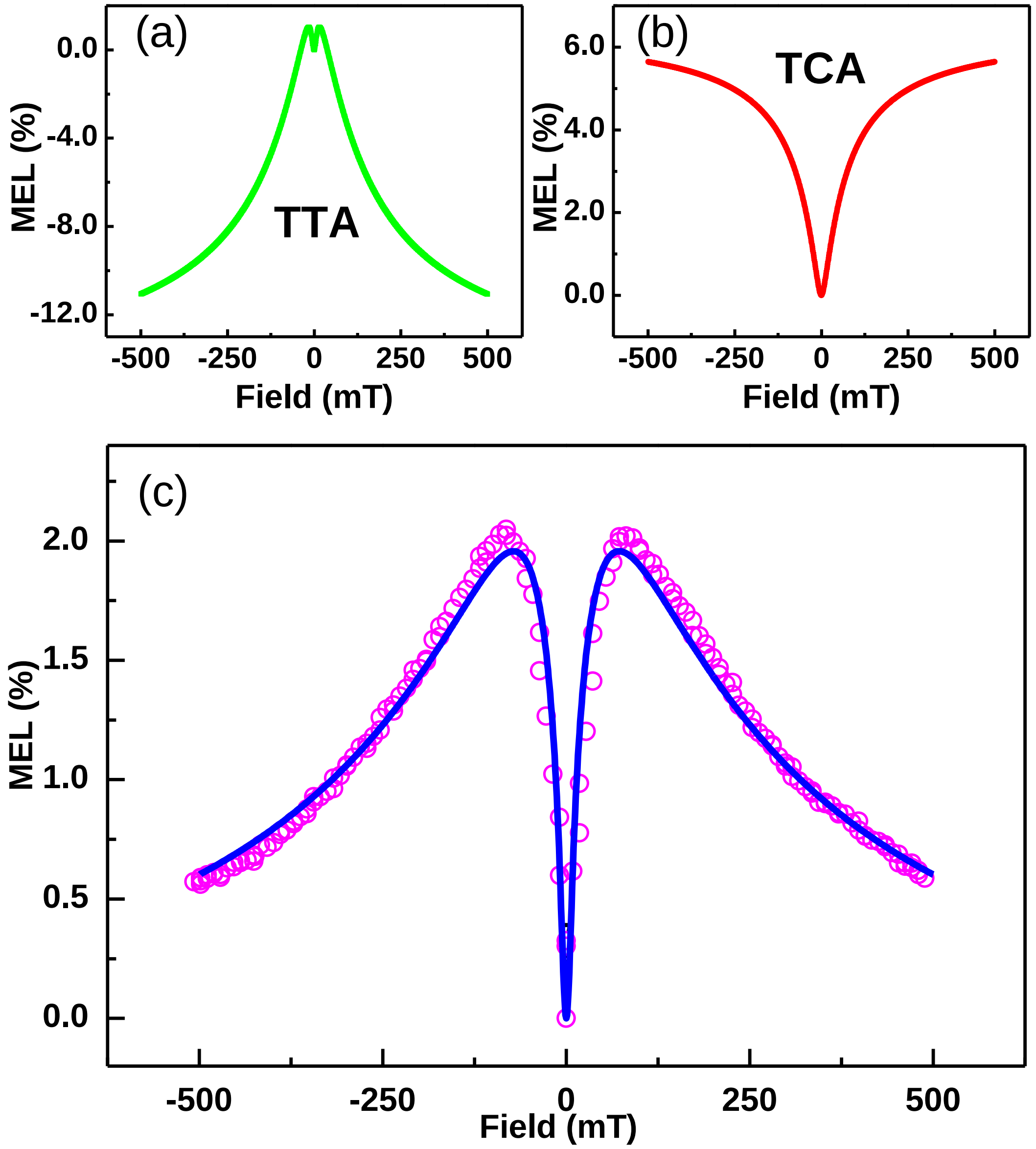

Figure. S2. Different parts of Figure 4(c): (a) MEL curve of TTA fraction, (b) MEL curve of TCA fraction, (c) experimental data (violet circle) and fitted result (blue curve).
